# Health-Related Quality of Life following Surgery for Native and Prosthetic Valve Infective Endocarditis

**DOI:** 10.3390/jcm11133599

**Published:** 2022-06-22

**Authors:** Shekhar Saha, Ralitsa Mladenova, Caroline Radner, Konstanze Maria Horke, Joscha Buech, Philipp Schnackenburg, Ahmad Ali, Sven Peterss, Gerd Juchem, Maximilian Luehr, Christian Hagl, Dominik Joskowiak

**Affiliations:** 1 Department of Cardiac Surgery, Ludwig Maximillian University of Munich, 81377 Munich, Germany; r.mladenova15@gmail.com (R.M.); caroline.radner@med.uni-muenchen.de (C.R.); konstanze.horke@med.uni-muenchen.de (K.M.H.); joscha.buech@med.uni-muenchen.de (J.B.); philipp.schnackenburg@med.uni-muenchen.de (P.S.); ahmad.ali@med.uni-muenchen.de (A.A.); sven.peterss@med.uni-muenchen.de (S.P.); gerd.juchem@med.uni-muenchen.de (G.J.); maximilian.luehr@uk-koeln.de (M.L.); christian.hagl@med.uni-muenchen.de (C.H.); dominik.joskowiak@med.uni-muenchen.de (D.J.); 2 German Centre for Cardiovascular Research (DZHK), Partner Site Munich Heart Alliance, 80802 Munich, Germany; 3 Department of Cardiothoracic Surgery, University Hospital Cologne, 50937 Cologne, Germany

**Keywords:** infective endocarditis, health-related quality of life, valve replacement

## Abstract

Objectives: The objective of this study was to compare the long-term outcomes and health-related quality of life (HRQOL) of patients following surgery for infective native valve endocarditis (NVE) and prosthetic valve endocarditis (PVE). Methods: We retrospectively identified 633 consecutive patients who had undergone surgery for infective endocarditis at our center between January 2005 and October 2018. The patients were interviewed, and the SF-36 survey was used to assess the HRQOL of survivors. Propensity score matching (2:1) was performed with data from a German reference population. Multivariable analysis incorporated binary logistic regression using a forward stepwise (conditional) model. Results: The median age of the cohort was 67 (55–74) years, and 75.6% were male. Operative mortality was 13.7% in the NVE group and 21.6% in the PVE group (*p* = 0.010). The overall survival at 1 year was 88.0% and was comparable between the groups. The physical health summary scores were 49 (40–55) for the NVE patients and 45 (37–52) for the PVE patients (*p* = 0.043). The median mental health summary scores were 52 (35–57) and 49 (41–56), respectively (*p* = 0.961). On comparison of the HRQOL to the reference population, the physical health summary scores were comparable. However, significant differences were observed with regard to the mental health summary scores (*p* = 0.005). Conclusions: Our study shows that there are significant differences in the various domains of HRQOL, not only between NVE and PVE patients, but also in comparison to healthy individuals. In addition to preoperative health status, it is important to consider the patient’s expectations regarding surgery. Further prospective studies are required.

## 1. Introduction

The incidence of infective endocarditis (IE) in Germany has been on the rise, with a case fatality rate of 17% [1]. The rising incidence of IE over the last decade may be attributed to several factors, which include an aging population, rise in the use of implantable cardiac devices, increase in the number of patients undergoing hemodialysis, and changes in antibiotic prophylaxis for the prevention of IE [1,2]. To date, long-term success following IE has primarily been described in relation to clinical criteria, such as cerebrovascular accidents, cardiac failure, need for cardiac surgery, relapse rate, and mortality [3]. These traditional criteria to assess the success of therapy no longer do justice to the increased interest of patients in maintaining a good quality of life, thus making the assessment of HRQOL even more important [4].

Following an episode of IE, patients have been reported to develop massive physical deconditioning and are at risk of developing anxiety and depression, as well as posttraumatic stress disorders [5]. This is reflected in our previous findings, where, following cardiac surgery, patients generally exhibited an improvement in health-related quality of life (HRQOL); however, in one-fifth of patients, there was no recovery of mental health status, even after 1 year [4]. Prosthetic valve endocarditis (PVE) has been reported to be associated with higher mortality rates than native valve endocarditis (NVE), whereas patients not undergoing surgery for IE have been reported to have mortality rates as high as 85% [6]. Although the current guidelines recommend surgery followed by antibiotics for the treatment of IE, the HRQOL of these patients has not been adequately investigated [7,8]. We analyzed the short-term and mid-term outcomes, as well as the HRQOL, of patients following surgical treatment for IE.

## 2. Patients and Methods

### 2.1. Ethics Statement

This study was approved by the ethics board of Ludwig Maximilian University (no. 19-730 and 20-821), and the requirement to obtain patient consent was waived for this retrospective study. Postoperative treatment and data acquisition were performed as part of routine patient care. Data acquisition was based on institutional databases and then de-identified. All procedures described in this study were in accordance with the institutional ethics boards and national data safety regulations.

### 2.2. Study Design and Definition of Groups

We retrospectively identified 663 consecutive patients who underwent cardiac surgery at our centre between January 2005 and October 2018. All patients consented to surgery; postoperative treatment and data acquisition were performed as part of routine patient care. Patient details were collected from our institutional database and de-identified. Additionally, the EuroSCORE II [9] was calculated, which predicts total perioperative mortality.

### 2.3. Definition of Parameters

Prosthetic valve endocarditis (PVE) was diagnosed according to the modified Duke’s criteria and the 2015 ESC guidelines on infective endocarditis [7].

Early PVE is defined as IE occurring within 1 year of surgery and late PVE as IE occurring beyond 1 year [7].

Reoperations were defined as one or more previous major cardiac operations involving opening the pericardium [9].

Adverse cerebrovascular events were defined as new-onset postoperative neurological symptoms accompanied by new computed tomography-confirmed central nervous system lesions [10].

Low cardiac output syndrome was defined as a constellation of mean arterial pressure < 60 mmHg, urine production < 0.5 mL/kg/h longer than 1 h, S_CV_O_2_ < 60% with SaO_2_ 98%, and serum lactate levels > 2.0 mmol/L [11].

Operative mortality was defined as in-hospital mortality and mortality within 30 days, regardless of cause.

### 2.4. Evaluation of Health-Related Quality of Life and Follow-Up

A survey was carried out for 557 survivors and included outpatient clinical records or data from telephone interviews with the general practitioner. HRQOL measurements were performed cross-sectionally. Patients were observed for a total of 2221 person-years, and the median follow-up time was 3.0 years (0.5–6.5 years).

To assess the HRQOL of the survivors, study participants were interviewed, and the SF-36 survey was used to evaluate the HRQOL of patients discharged from the hospital, as previously described [12,13]. The SF-36 questionnaire was sent out to all survivors. Complete data were available for 229 patients. The following areas were evaluated: physical functioning (PF), role physical (RP), bodily pain (BP), general health (GH), vitality (VT), social functioning (SF), role emotional (RE), and mental health (MH). Based on these scores, a summary physical score (PHS) and a summary mental health score (MHS) were calculated. Furthermore, the results were compared to a German reference population, as described below.

### 2.5. Data Collection and Statistical Analysis

Data were analyzed using IBM SPSS version 25 (Statistical Package for the Social Sciences) (IBM-SPSS Inc., Armonk, NY, USA). Data were tested for normal distribution using the Kolmogorov–Smirnov test with Lilliefors correction. Categorical variables were evaluated using chi-square and Fisher‘s exact tests, and continuous variables were evaluated using the Mann–Whitney *U* test. Survival analysis was performed with Kaplan–Meier curves and log-rank tests. All analyses were two-tailed. The null hypothesis was rejected, and significant difference was assumed at *p*-values < 0.05. Data are presented as medians (25–75th quartiles) or absolute values (percentages), unless otherwise specified. To compensate for the differences between the standard control population, a propensity score matching analysis was performed. For this purpose, logistic regression was used to develop a propensity score. A propensity score difference of 0.05 was used as a maximum caliper for matching the two groups. The patients were matched with healthy controls in a 1:2 manner based on age and gender.

### 2.6. Data Availability Statement

The data underlying this study cannot be shared publicly, in accordance with national data safety guidelines, to protect the privacy of individuals included in the study. The data will be shared on reasonable request to the corresponding author.

## 3. Results

### 3.1. Baseline Parameters

Patient characteristics and baseline parameters are outlined in Table 1. The median EuroSCORE II was 23.2% (14.7–36.3%) in the NVE group and 51.5% (39.0–67.2%) in the PVE group (*p* < 0.001). The majority of the patients were male, with a higher number of males in the PVE group (341 (72.1%) vs. 160 (84.2%), *p* = 0.001). Higher rates of arterial hypertension (297 (62.8%) vs. 151 (79.5%), *p* < 0.001), hyperlipoproteinemia (116 (24.5%) vs. 102 (53.7%), *p* < 0.001), coronary artery disease (138 (29.2%) vs. 76 (40.0%), *p* = 0.007), previous pacemaker (13 (2.8%) vs. 23 (12.1%), *p* < 0.001), and smoking (96 (20.3%) vs. 52 (27.4%), *p* = 0.048) were observed in the PVE group. In this cohort, the main causative organisms were Streptococcus species (*n* = 167 (25.2%)), Staphylococcus aureus (*n* = 154 (23.3%)), Enterococcus species (*n* = 89 (13.4%)), other Staphylococci (*n* = 65 (9.8%)), Propionibacterium species (*n* = 12 (1.8%)), and HACEK organisms (*n* = 8 (1.2%)). Blood-culture-negative infective endocarditis was diagnosed in 103 patients (15.5%).

### 3.2. Outcomes

Data on the main morbidities and outcomes are listed in Table 2. Higher rates of tracheostomy (30 (6.3%) vs. 23 (12.1%), *p* = 0.012), postoperative pacemaker implantation (31 (6.6%) vs. 48 (25.3%), *p* = 0.016), septic shock (81 (17.1%) vs. 50 (10.6%), *p* = 0.007), and ECLS support (19 (4.0%) vs. 29 (15.3%), *p* < 0.001) were observed in the PVE group. The length of hospital stay (16 days (8–26 days) vs. 20 days (12–34 days), *p* = 0.001) and length of ICU stay (4 days (2–7 days) vs. 5 days (3–12 days), *p* < 0.001) were longer in the PVE group. Operative mortality was 13.7% in the NVE group and 21.6% in the PVE group (*p* = 0.010).

### 3.3. Quality of Life and Follow-Up

**Figure 1:**
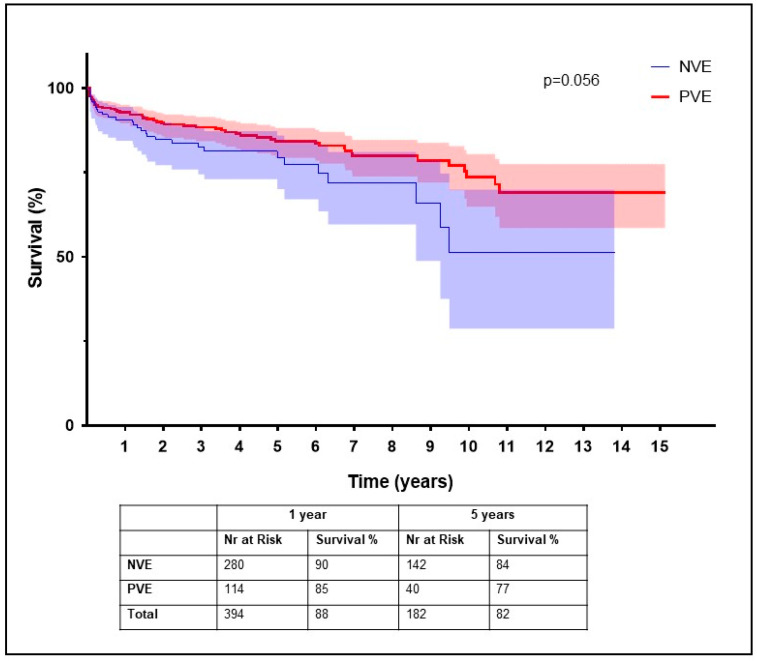
Kaplan–Meier survival curves for native valve endocarditis versus prosthetic valve endocarditis.

Among the 557 survivors, 25 patients (4.5%) abstained from participating in the survey. A total of 68 patients died during the follow-up period and 235 were lost to follow-up. Data on HRQOL were available for a total of 229 patients. The median scores of the study cohort for the eight subscale categories and summary scores for the NVE, PVE, and control groups are illustrated in Table 3. There were significant differences in the domains of physical functioning (*p* = 0.005), role physical (*p* < 0.001), bodily pain (*p* = 0.001), social functioning (*p* < 0.001), and role emotional (*p* < 0.001). Furthermore, we observed a significant difference in the mental health summary score (*p* = 0.005). Details of the individual domains are provided in Table 3. Survival at 1 year was 90% in the NVE group and 85% in the PVE group, and survival at 5 years was 84% in the NVE group and 77% in the PVE group (*p* = 0.056) (Figure 1).

## 4. Discussion

Patients suffering from IE are a heterogeneous cohort, which includes those who are successfully treated with no adverse events and those with severe complications and a high rate of mortality [14]. As mentioned earlier, the conventional criteria of absence of morbidities and mortality are not enough to determine the success of treatment, due to the increased interest of patients in maintaining a good quality of life [4]. There are several tools available to assess quality of life, and for the purpose of this study, we evaluated HRQOL using the SF-36 survey, a well-established instrument for the assessment of HRQOL. Our analysis revealed that hospital survivors had significantly different summary scores for physical health and mental health, especially older patients and those suffering from PVE. 

Forestier et al. [15] reported that in older patients, any infection, especially IE, may severely impair functional and cognitive capacities and result in long-lasting disability. Immunosenescence and multiple comorbidities render older patients susceptible to IE [15,16]. Furthermore, major factors that determine postoperative outcome are the disease-independent influence of the biological aging process and, therefore, the cellular and tissue aging process, in addition to the presence of comorbidities such as advanced atherosclerosis, impaired diastolic heart function, renal insufficiency, reduced lung compliance, and respiratory muscle strength [17,18]. Nutritional and functional status have also been found to be independent predictors of mortality in older patients suffering from IE [15]. Complex valve surgery, even in the setting of reoperation in older patients, has been reported to be feasible with good outcomes [18,19]. Although cardiac surgery in elderly patients has been reported to be associated with an improvement in HRQOL, a decline in HRQOL has been reported in about 8–19% of patients [20]. In patients suffering from IE, it is alarming to see that older patients fare worse than younger patients in six out of eight domains of HRQOL and record significantly lower physical health summary scores.

### 4.1. HRQOL following IE and Comparison to the Standard Population

When compared to NVE, PVE remains a serious condition with considerably higher in-hospital mortality of 19–50% versus 7–13% in NVE patients [21]. In our cohort, patients following PVE had comparable mid-term outcomes; however, there were significant differences in physical functioning, role physical, bodily pain, and the mental health summary scores. The poor outcomes may be strongly related to the complicated clinical course in the setting of a reoperation, but also to the critical preoperative state, which may prolong the recovery of HRQOL.

Perrotta et al. [22] also assessed HRQOL using the SF-36 and found no significant differences in the scales for physical and mental health when compared to a healthy age- and gender-matched control group. In contrast, our results indicated significant differences in the physical subdomains as well as in the mental health summary scores. Although survival following surgery for IE has improved over the years, HRQOL has remained unaddressed [8]. HRQOL measures are based on how patients perceive and experience the aftermath of surgery in their daily lives. 

### 4.2. HRQOL and Neurological Sequelae

Neurological complications have been reported to occur in 20–40% of patients suffering from IE [23]. In our cohort, preoperative cerebral emboli were diagnosed in almost one-fourth of the patients. Additionally, another one-fourth of the patients suffered from postoperative adverse cerebrovascular events. Stroke survivors have been reported to enjoy a good quality of life; however, it is important to assist stroke survivors in coping, as well as maintaining and strengthening their support systems.

Following discharge, it has been reported that about one-third of IE patients suffer from concentration problems and memory loss, about half from fatigue, and about three-fourths from physical weakness [24]. Furthermore, it has been reported that 35% of previously employed patients had not returned to work after 1 year following an episode of IE. Following IE, patients also develop a negative perception of health, up to 55% experience anxiety and depression, and 11% have been reported to exhibit signs of post-traumatic stress disorder [3,24].

### 4.3. Perspectives in Patient Care

The phenomenon that patients assess their health and HRQOL as equal or even better, especially following serious health events, can be explained by an adaptation process referred to as a “response shift” [25]. This describes how individuals revise their health standards or their priorities when suffering a significant deterioration in their objective health status [26]. Behavioral and lifestyle patterns, such as sedentary behavior, poor adherence to medication, diet, exercise, and smoking cessation, as well as a higher body mass index, high blood pressure, hyperlipidemia, and diabetes, may be attributable to emotional distress [4]. In our cohort, we saw significantly higher rates of these comorbidities in the PVE group. This too may be a factor that negatively impacts recovery and HRQOL.

Microorganisms are also suspected to play a role in the progress of the disease. Relatively avirulent microorganisms, such as viridans streptococci, Streptococcus bovis, or HACEK species (*Haemophilus* species, *Actinobacillus actinomycetemcomitans*, *Cardiobacterium hominis*, *Eikenella* species, and *Kingella kingae*), require a longer time for the progression of the disease as compared to more virulent microorganisms, such as Staphylococcus aureus [3]. The gradual worsening of the symptoms may lead to altered perception of HRQOL following surgery for IE.

Considering the relatively poor HRQOL in both physical and mental spheres following surgery for IE, cardiac rehabilitation may be an important adjunctive therapy. Although evidence for its role in IE is lacking, cardiac rehabilitation has been reported to be advantageous for patients suffering from coronary artery disease and heart failure [24]. In addition to exercise training, interventions such as patient education have been shown to improve HRQOL and decrease healthcare costs, and psychological support has been shown to improve psychological symptoms, such as depression and anxiety [24]. Furthermore, cognitive appraisal of the significance of illness and the ability to cope with stressful events are important to ensure a good quality of life, and may be improved through rehabilitation measures [27]. Furthermore, it is important to keep in mind that patients who refer to themselves as “healthier” have higher treatment expectations, and this may result in negative experiences adversely affecting recovery and self-perceived HRQOL after surgery [4].

Endocarditis teams achieve better compliance in antimicrobial therapy and fewer cases of renal failure, deaths by embolic events, and multiple organ failure [28]. Furthermore, studies have shown their impact, with improvements in early diagnosis, management strategies, and survival [16]. Further studies on HRQOL are required to procure information on the impact of interventions and cardiac operations, not only to justify the decision to operate, but also to be able to help patients make informed decisions.

## 5. Conclusions

Our study shows that there are significant differences in the various domains of HRQOL, not only between NVE and PVE groups, but also in comparison to healthy individuals. Despite adequate surgical therapy, prosthetic endocarditis remains one of the most serious complications in the treatment of valvular heart disease, decisively affecting both somatic health and quality of life. In addition to preoperative health status, it is important to consider the patient’s expectations regarding surgery. Further prospective studies on the prevention of prosthetic endocarditis are needed.

## 6. Limitations

This was a retrospective single-center study with the inherent limitation of such an analysis. The small number of patients at follow-up is associated with low power of statistical analyses. As this was a descriptive retrospective registry of patients operated on for IE, HRQOL measurements were taken at different time points after their surgery. Furthermore, the analysis of variables was limited to a univariable analysis due to the sample size. Further studies with longer follow-up are required.

## Figures and Tables

**Table 1:** **Baseline parameters.** Data are presented as medians (25–75th percentiles) or absolute numbers (percentages). BMI: body mass index, COPD: chronic obstructive pulmonary disease, LVEF: left ventricular ejection fraction, and PVE: prosthetic valve endocarditis.

	NVE (*n* = 473)	PVE (*n* = 190)	*p*-Value
Age (years)	64 (52–74)	69 (60–75)	<0.001
BMI (kg/m^2^)	24.7 (22.6–23.6)	25.6 (23.6–27.8)	0.033
BMI > 25 kg/m^2^	223 (47.1)	106 (55.8)	0.039
Male (%)	341 (72.1)	160 (84.2)	0.001
EuroSCORE II (%)	23.2 (14.7–36.3)	51.5 (39.0–67.2)	<0.001
NYHA class			0.005
NYHA I–II	159 (33.6)	43 (22.6)	
NYHA III–IV	314 (66.4)	147 (77.4)	
LVEF			0.018
≥50%	201 (42.5)	65 (34.2)	
31–49%	251 (53.1)	107 (56.3)	
≤30%	21 (4.4)	18 (9.5)	
Arterial hypertension (%)	297 (62.8)	151 (79.5)	<0.001
Hyperlipoproteinemia (%)	116 (24.5)	102 (53.7)	<0.001
Chronic kidney disease (%)	162 (34.2)	70 (36.8)	0.527
Dialysis (%)	25 (5.3)	14 (7.4)	0.303
Creatinine clearance (mL/min)	63 (42–87)	53 (39–69)	<0.001
Creatinine clearance < 50 mL/min	165 (34.9)	85 (44.7)	0.018
Smokers (%)	96 (20.3)	52 (27.4)	0.048
COPD (%)	46 (9.7)	28 (14.7)	0.061
Coronary artery disease (%)	138 (29.2)	76 (40.0)	0.007
Diabetes mellitus (%)	88 (18.6)	45 (23.7)	0.140
Pacemaker (%)	13 (2.8)	23 (12.1)	<0.001
Peripheral vascular disease (%)	34 (7.2)	17 (9.0)	0.425
Prior neurological disorder (%)	116 (24.5)	52 (27.4)	0.552
Preoperative cerebral emboli (%)	107 (22.6)	48 (25.3)	0.456
Intravenous drug use (%)	8 (1.7)	3 (1.6)	0.922
Reoperations (%)	29 (6.1)	184 (96.8)	<0.001
Time to PVE (years)	-	3.0 (0.6–7.6)	-
Early PVE (%)	-	47 (7.1)	-
Vegetations (%)	355 (75.1)	149 (78.4)	0.455

**Table 2:** **Postoperative complications and outcomes.** Data are presented as medians (25–75th percentiles) or absolute numbers (percentages). ECLS: extracorporeal life support, IABP: intra-aortic balloon pump, ICU: intensive care unit, LCOS: low cardiac output syndrome, and PMV: postoperative mechanical ventilation.

Morbidities	NVE (*n* = 473)	PVE (*n* = 190)	*p*-Value
Adverse cerebrovascular events (%)	105 (22.3)	54 (28.4)	0.106
Severe bleeding with re-exploration (%)	47 (10.0)	35 (18.4)	0.003
Tracheostomy (%)	30 (6.3)	23 (12.1)	0.012
Pacemaker implantation (%)	31 (6.6)	48 (25.3)	0.016
Renal replacement therapy (%)	39 (8.2)	23 (12.1)	0.123
LCOS (%)	51 (10.8)	30 (15.8)	0.088
Septic shock (%)	81 (17.1)	50 (10.6)	0.007
ECLS support (%)	19 (4.0)	29 (15.3)	<0.001
IABP (%)	9 (1.9)	5 (2.6)	0.544
**Outcomes**			
Operative mortality (%)	65 (13.7)	41 (21.6)	0.010
Length of hospital stay (days)	16 (8–26)	20 (12–34)	0.001
Length of ICU stay (days)	4 (2–7)	5 (3–12)	<0.001
ICU stay > 14 days (%)	53 (11.2)	42 (22.1)	0.001
ICU stay > 7 days (%)	137 (30.0)	72 (38.0)	0.025
Length of PMV (hours)	13 (8–29)	20 (12–34)	<0.001
**Follow-up**			
Recurrence (%)	33 (7.0)	17 (8.9)	0.389
Redo surgery for endocarditis (%)	23 (4.9)	5 (2.6)	0.195
Any surgical procedure (%)	60 (12.7)	27 (14.2)	0.606

**Table 3:** Results of the SF-36 survey. NVE: native valve endocarditis and PVE: prosthetic valve endocarditis. Data are presented as medians (25–75th percentiles).

	NVE (*n* = 160)	PVE (*n* = 69)	Standard (*n* = 458)	*p* ^a^
Physical functioning	80 (63–95)	75 (50–90)	90 (60–95)	0.005
Role physical	100 (25–100)	63 (0–100)	100 (50–100)	<0.001
Bodily pain	100 (62–100)	84 (62–100)	74 (51–100)	0.001
General health	62 (46–72)	59 (39–77)	62 (45–77)	0.600
Vitality	60 (40–75)	55 (40–70)	60 (45–75)	0.135
Social functioning	88 (63–100)	88 (63–100)	100 (75–100)	<0.001
Role emotional	100 (33–100)	100 (33–100)	100 (100–100)	<0.001
Mental health	76 (64–88)	76 (64–84)	76 (64–88)	0.678
Physical summary score	49 (40–55)	45 (37–52)	49 (36–55)	0.315
Mental summary score	52 (35–57)	49 (41–56)	54 (49–58)	0.005

^a^ Kruskal–Wallis test.

## Data Availability

The data presented in this study are available on reasonable request from the corresponding author. The data are not publicly available in accordance to national data safety guidelines.

## Abbreviations

BP	bodily pain
ECLS	extracorporeal life support
EuroSCORE II	European System for Cardiac Operative Risk Evaluation II
GH	general health
HRQOL	health-related quality of life
IABP	intra-aortic balloon pump
IE	infective endocarditis
MH	mental health
MHS	mental health score
NVE	native valve endocarditis
PF	physical functioning
PHS	physical health score
PVE	prosthetic valve endocarditis
RE	role emotional
RP	role physical
SF	social functioning
SF-36	Short Form 36
VIT	vitality
